# Interpretable Machine Learning Algorithms to Predict the Axial Capacity of FRP-Reinforced Concrete Columns

**DOI:** 10.3390/ma15082742

**Published:** 2022-04-08

**Authors:** Celal Cakiroglu, Kamrul Islam, Gebrail Bekdaş, Sanghun Kim, Zong Woo Geem

**Affiliations:** 1 Department of Civil Engineering, Turkish-German University, Istanbul 34820, Turkey; cakiroglu@tau.edu.tr; 2 Department of Civil, Geological and Mining Engineering, Polytechnique Montréal, Montreal, QC H3C 3A7, Canada; kamrul.islam@polymtl.ca; 3 Department of Civil Engineering, Istanbul University—Cerrahpasa, Istanbul 34320, Turkey; 4 Department of Civil and Environmental Engineering, Temple University, Philadelphia, PA 19122, USA; sanghun.kim@temple.edu; 5 Department of Smart City & Energy, Gachon University, Seongnam 13120, Korea

**Keywords:** fiber-reinforced polymer (FRP) rebar, reinforced concrete columns, axial capacity, machine learning, ensemble learning, harmony search optimization

## Abstract

Fiber-reinforced polymer (FRP) rebars are increasingly being used as an alternative to steel rebars in reinforced concrete (RC) members due to their excellent corrosion resistance capability and enhanced mechanical properties. Extensive research works have been performed in the last two decades to develop predictive models, codes, and guidelines to estimate the axial load-carrying capacity of FRP-RC columns. This study utilizes the power of artificial intelligence and develops an alternative approach to predict the axial capacity of FRP-RC columns more accurately using data-driven machine learning (ML) algorithms. A database of 117 tests of axially loaded FRP-RC columns is collected from the literature. The geometric and material properties, column shape and slenderness ratio, reinforcement details, and FRP types are used as the input variables, while the load-carrying capacity is used as the output response to develop the ML models. Furthermore, the input-output relationship of the ML model is explained through feature importance analysis and the SHapely Additive exPlanations (SHAP) approach. Eight ML models, namely, Kernel Ridge Regression, Lasso Regression, Support Vector Machine, Gradient Boosting Machine, Adaptive Boosting, Random Forest, Categorical Gradient Boosting, and Extreme Gradient Boosting, are used in this study for capacity prediction, and their relative performances are compared to identify the best-performing ML model. Finally, predictive equations are proposed using the harmony search optimization and the model interpretations obtained through the SHAP algorithm.

## 1. Introduction

Fiber-reinforced polymers (FRP) have gained popularity in construction as a viable replacement for steel rebars in reinforced concrete (RC) members due to their corrosion resistance, higher strength, lightweight, and ease of fabrication [1]. Extensive research works have been carried out in the last two decades to investigate the performance of the different types of FRP reinforcement [2,3,4,5,6,7,8,9,10]. Some of the studies in this area were experimental in nature [11,12,13,14,15,16,17,18,19], whereas others aimed to develop more accurate, novel methods of prediction for structural performance using various techniques of artificial intelligence and optimization [20,21,22,23,24,25]. Murad et al. [23] used gene expression programming on FRP-RC beams to predict flexural strength. As the variables affecting the flexural strength, the beam cross-sectional dimensions, concrete compressive strength, the area, the elasticity modulus, and the ultimate tensile strength of the FRP reinforcement were selected. An experimental database consisting of 116 samples was used to develop the predictive model. Protchenko et al. [24] used an experimental database composed of 102 samples to further improve the existing predictive models in the ACI 440.1R-15 code pertaining to the flexural capacity of FRP-RC beams. While a relatively large amount of research in the literature deals with the experimental study of FRP-RC columns, the literature that deals with the applications of machine learning techniques for the predictive modeling of these structures is somewhat limited. Raza et al. [22] developed a large database of 279 specimens of GFRP-RC columns to develop an improved, ANN-based predictive model to estimate the axial capacity. Bakouregui et al. [25] applied the eXtreme Gradient Boosting (XGBoost) algorithm for the prediction of the axial load-carrying capacity of FRP-reinforced concrete columns. An experimental database consisting of 283 samples was chosen to train the model. The model output was interpreted using the SHAP algorithm to determine the effects of the various input parameters on the model output. In addition to the load eccentricity, the gross cross-sectional area, concrete compressive strength, column slenderness ratio, and the spacing between the transverse reinforcements were found to have the greatest impact on the axial load-carrying capacity of FRP-RC columns.

The current study focuses on the concentric loading case. In addition to the XGBoost algorithm, seven other ML algorithms are applied to a portion of the database compiled by Bakouregui et al. [25], which consists of samples with concentric loading. One of the objectives of the current study is to perform a systematic comparison between different ensemble ML models to predict the compressive capacity of FRP-RC columns, which is missing in the existing literature. The relative computational efficiency of the different ML algorithms is presented as well. To fulfill the objective, we select 117 FRP-RC column test specimens from an existing database [25]. The existing database consists of 283 FRP-RC column test specimens that include both short and slender column specimens subjected to concentric and eccentric loading. The other objective of the current study is to develop opensource, Python-based ML models to estimate the axial load-carrying capacity of FRP-RC columns to further help researchers utilize the developed models to improve the framework once additional experimental data are available.

The input variables of the database used in this paper are the slenderness ratio, gross cross-sectional area, the type of cross-section (circular or rectangular), the type of concrete (light-weight or normal-weight concrete), compressive strength of concrete, type of composite material used in the longitudinal reinforcement (GFRP or CFRP), longitudinal reinforcement ratio, elasticity modulus of the longitudinal reinforcements, the ultimate strength of the longitudinal reinforcements, type of the transverse reinforcement material (GFRP, CFRP, or steel), the configuration of transverse reinforcement (i.e., spirals or ties), and the spacing of the transverse reinforcement. The output variable is the axial load-carrying capacity of the corresponding specimen. The database is split into a training and test set in 90% to 10% ratio, and the performances of the different machine learning models are compared using four different accuracy metrics. Afterward, the SHAP algorithm is utilized to determine the most significant input variables and their interdependencies. Three different, closed-form equation formats are proposed based on the outcome of the SHAP algorithm. All three of these equations depend on a certain number of parameters that can be optimized to minimize the difference between the equation output and the actual axial load-carrying capacity measurements. A well-established, metaheuristic optimization technique called harmony search optimization is applied in this optimization process. Then, the performances of these two equations are compared using different accuracy metrics. The current study is unique in its attempt to obtain closed-form equations for the prediction of the axial load-carrying capacity of FRP-RC columns based on ensemble machine learning algorithms, the SHAP procedure, and metaheuristic optimization. Relatively high accuracies were observed from the obtained equations. The following sections further elaborate on the process of obtaining predictive models for these structures based on experimental data.

## 2. Machine Learning and Optimization Methodologies

**Figure 1:**
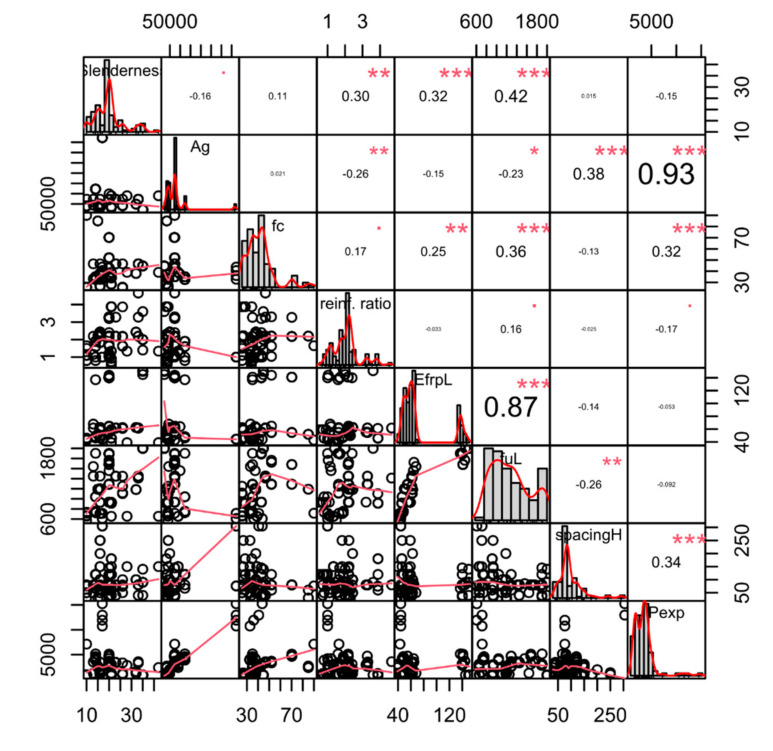
Correlation matrix of the dataset.

Eight data-driven machine learning models were applied to the database consisting of 117 samples. Each of these samples contained seven continuous-valued input variables, five discrete-valued input variables, and one continuous-valued output variable, the axial load-carrying capacity. The statistical distribution of these variables is shown in Figure 1.

Figure 1 shows the correlation plot for the seven non-categorical, continuous-valued input variables together with the output variable Pexp. Ag, *f_c_*’, *E_FRP_*, fuL, spacingH, and Pexp stand for the gross cross-sectional area, concrete compressive strength at 28 days, elasticity modulus of the longitudinal reinforcements, the ultimate strength of the longitudinal reinforcements, spacing between transverse reinforcements, and the experimental axial load-carrying capacity, respectively. A list of all the variable names and abbreviations used in this paper can be found in Appendix A. In Figure 1, for each variable on a diagonal tile, the scale of this variable is shown either on the left vertical or the right vertical axis. In addition to the vertical axes, for each variable, the same scaling is shown on one of the horizontal axes as well. The lower triangle in this plot contains bivariate scatter plots with regression lines, while the diagonal contains histograms showing the distributions of all variables. The upper triangle contains the Pearson correlation coefficients (r_xy_) which can be computed using Equation (1). In Equation (1), x and y are two sequences of variable values between which the Pearson correlation is being computed. A Pearson correlation value close to 1 indicates a high correlation between the two variables. The significance of the correlation is indicated with stars in the upper triangle where the number of stars is proportional to the level of significance. According to Figure 1, the gross cross-sectional area Ag has the greatest correlation with Pexp followed by the transverse reinforcement spacing and the concrete compressive strength. The remaining continuous-valued input variables are negatively correlated to Pexp.

(1) $$r_{\mathrm{xy}}=\frac{n\sum_{i=1}^{n}x_{i}y_{i}-\sum_{i=1}^{n}x_{i}\sum_{i=1}^{n}y_{i}}{\sqrt{n\sum_{i=1}^{n}x_{i}^{2}-{\left(\sum_{i=1}^{n}x_{i}\right)}^{2}}\sqrt{n\sum_{i=1}^{n}y_{i}^{2}-{\left(\sum_{i=1}^{n}y_{i}\right)}^{2}}}$$

In what follows, the machine learning models applied in this study are briefly presented.

### 2.1. Machine Learning Models

All models presented in this section were trained and tested using both the continuous-valued and discrete-valued input variables. The discrete-valued input variables, namely, the type of cross-section, type of concrete, type of material used in the longitudinal and transverse reinforcements, and the configuration of the transverse reinforcements were one-hot encoded before being used in the training and testing of the machine learning models. The machine learning models applied in this study are briefly presented as follows. For further details of the algorithms presented in this section, it is suggested that the reader checks the references [26,27,28,29,30,31,32,33,34,35,36,37].

#### 2.1.1. Kernel Ridge Regression (KRR)

Let $\left(P_{n},x_{n}\right)\in \mathbb{R}\times {\mathbb{R}}^{17}$, where $x_{n}$ is the n-th training sample containing the information of 17 one-hot encoded and continuous input variables. Let **x** be any sample outside of the training set. The kernel ridge prediction $\hat{P}$ for this new sample is calculated using Equation (2) where κ is a kernel function, and N is the total number of samples in the training set [26].

(2) $$\hat{P}=\overset{N}{\underset{n=1}{\sum }}{\hat{\theta }}_{n}\kappa \left(x,x_{n}\right)$$

The coefficients ${\hat{\theta }}_{n}$ of the kernel ridge regressor are computed using Equation (3), where C is the regularization constant, κ is the kernel matrix, **I** is the identity matrix, $\hat{\theta }={\left[{\hat{\theta }}_{1},\;\ldots ,{\hat{\theta }}_{N}\right]}^{T}$, and $P={\left[P_{1},\;\ldots ,{\;P}_{N}\right]}^{T}$.

(3) $$\left(\kappa +CI\right)\hat{\theta }=P$$

#### 2.1.2. Support Vector Regression (SVR)

The Support Vector Regression technique aims to reduce the computational complexity by limiting the training operations to a subset of the training set. Equation (4) shows the predictive model for a vector **x** from outside the training set [27].

(4) $$\hat{P}=\overset{N}{\underset{n=1}{\sum }}\left(a_{n}-{\hat{a}}_{n}\right)\kappa \left(x,x_{n}\right)+b$$

In Equation (4), $a_{n}$ and ${\hat{a}}_{n}$ are the Lagrange multipliers that satisfy the conditions $0\leq a_{n}\leq C$ and $0\leq {\hat{a}}_{n}\leq C$. The regularization parameter C works towards reducing the noise in the training set as the smaller values of C correspond to greater regularization. In Equation (4), only the training samples for which $a_{n}-{\hat{a}}_{n}\neq 0$ have an impact on the model output, and they are called the support vectors.

#### 2.1.3. Lasso Regression

The Lasso Regression technique is based on the linear predictive model shown in Equation (5) [28].

(5) $$y\left(x\right)=\beta_{0}+\overset{17}{\underset{n=1}{\sum }}\beta_{n}x_{n}$$

The model coefficients $\beta_{n}$ in Equation (5) are estimated through the minimization of the objective function shown in Equation (6). The second term in Equation (6), calculating the L1 norm of the coefficient vector, leads to some of the model coefficients associated with the less significant input variables being set to zero. This yields a predictive model that depends on a smaller number of variables which can also be called a sparse model [28].

(6) $$\overset{N}{\underset{i=1}{\sum }}{\left(P_{i}-\beta_{0}-\overset{17}{\underset{j=1}{\sum }}\beta_{j}x_{\mathrm{ij}}\right)}^{2}+\lambda \overset{17}{\underset{j=1}{\sum }}\left|\beta_{j}\right|$$

#### 2.1.4. Gradient Boosting Machine (GBM)

GBM is based on the idea of consecutively fitting models to data to increase the model accuracy so that the new base learners $h\left(x,\theta \right)$ have maximum correlation with the negative gradient $g_{t}\left(x\right)$ of the loss function $\psi \left(y,f\right)$ [29]. In every iteration of the GBM algorithm, the best gradient descent step $\rho_{t}$ is calculated as in Equation (7), and the estimator function is updated as in Equation (8) [29,30].

(7) $$\rho_{t}=\arg\min_{\rho }\overset{N}{\underset{i=1}{\sum }}\psi \left[y_{i},{\hat{f}}_{t-1}\left(x_{i}\right)+\rho h\left(x_{I},\theta_{t}\right)\right]$$

(8) $${\hat{f}}_{t}:={\hat{f}}_{t-1}+\rho_{t}h\left(x,\theta_{t}\right)$$

#### 2.1.5. Adaptive Boosting (AdaBoost)

AdaBoost is an ensemble learning technique that iteratively runs weak regressors $h_{t}\left(x\right)$ and merges them to produce a strong regressor. The procedure for obtaining the final strong regressor $h_{f}\left(x\right)$ is given in Equations (9) and (10), where $\varepsilon_{t}$ is the loss associated with $h_{t}\left(x\right)$ [31].

(9) $$h_{f}\left(x\right)=\inf\left\{y\in Y:\;\underset{t:h_{t}\left(x\right)\leq y}{\sum }\log\left(\frac{1}{\beta_{t}}\right)\geq \frac{1}{2}\underset{t}{\sum }\log\left(1/\beta_{t}\right)\right\}$$

(10) $$\beta_{t}=\frac{\varepsilon_{t}}{1-\varepsilon_{t}}$$

#### 2.1.6. Random Forest (RF)

Random Forest is another ensemble learning algorithm that merges the output of decision tree models trained on subsets of the training set for a more accurate prediction. In addition to the random subsampling of the training set for the training of the decision trees, in the node splitting phase while generating a tree, a randomly selected subset of the input variables is utilized. The format of the Random Forest model is given in Equation (11), where ${\hat{m}}_{j}$ represents one of the decision trees [32,33].

(11) $$\hat{m}\left(x\right)=\frac{1}{M}\overset{N}{\underset{i=1}{\sum }}{\hat{m}}_{j}\left(x\right)$$

#### 2.1.7. Extreme Gradient Boosting (XGBoost)

XGBoost is a tree boosting algorithm that differentiates itself through high scalability and computational speed. The algorithm is capable of scaling to billions of samples in distributed settings. The output of the XGBoost model can be summarized as in Equation (12), where f_k_ denotes a regression tree, and K is the total number of regression trees [25,34]. The optimum values of the leaf weights $w_{j}^{*}$ that minimize the loss can be computed as in Equation (13), where l denotes the loss function, and *I_j_* is the set that contains the indices of the data samples associated with the j-th leaf [35].

(12) $${\hat{y}}_{i}=\overset{K}{\underset{k=1}{\sum }}f_{k}\left(x_{i}\right)$$

(13) $$w_{j}^{*}=-\frac{\sum_{i\in I_{j}}g_{i}}{\sum_{i\in I_{j}}h_{i}+\lambda },{\;g}_{i}=\frac{\partial l\left(y_{i},{\hat{y}}_{i}^{\left(t-1\right)}\right)}{\partial {\hat{y}}_{i}^{\left(t-1\right)}},\;\;g_{i}=\frac{\partial^{2}l\left(y_{i},{\hat{y}}_{i}^{\left(t-1\right)}\right)}{\partial {\left({\hat{y}}_{i}^{\left(t-1\right)}\right)}^{2}}\;$$

#### 2.1.8. Categorical Gradient Boosting (CatBoost)

CatBoost is another gradient boosting algorithm that performs particularly well in the presence of categorical data. In addition to the better processing of categorical features, the implementation of ordered boosting contributes to better accuracy in this algorithm. CatBoost addresses a particular issue called prediction shift, which is present in other gradient boosting algorithms such as XGBoost [36,37]. CatBoost overcomes the issue of prediction shift through the implementation of ordered boosting with ordered target statistics, the details of which can be found in [37].

### 2.2. Harmony Search Optimization

Metaheuristic algorithms have been applied to a large number of engineering problems in recent years. These algorithms are shown to effectively address problems characterized by high nonlinearity or non-differentiability [38]. One of the most widely used and well-established algorithms in this category is the harmony search algorithm. The algorithm was invented by Geem et al. [39] and has been applied to a wide range of engineering problems such as the optimum design of retaining walls [40,41], laminated composite plates [42,43], highway bridge plate girders [44], concrete-filled steel tubular columns [45], reinforced concrete cylindrical walls [46,47,48], and tuned liquid dampers [49].

The algorithm starts with the generation of an arbitrarily sized population consisting of optimum-solution candidate vectors, also called harmony vectors (HV). The number of harmony vectors in this population is called the harmony memory size (HMS). Each harmony vector has a size equal to the number of parameters being optimized. After the generation of the initial population, all harmony vectors go through the harmony search iterations. These iterations are defined by Equations (14)–(17).

(14) $$k=\mathrm{int}\left(\mathrm{rand}\cdot \mathrm{HMS}\right),\;\mathrm{rand}\in \left(0,1\right)$$

(15) $$\begin{array}{c} x_{i,\mathrm{new}}=x_{i,\min}+\mathrm{rand}\cdot \left(x_{i,\max}-x_{i,\min}\right),\\ \mathrm{if}\;\mathrm{HMCR}\;>\;\mathrm{rand} \end{array}$$

(16) $$\begin{array}{c} x_{i,\mathrm{new}}=x_{i,k}+\mathrm{rand}\cdot \mathrm{PAR}\cdot \left(x_{i,\max}-x_{i,\min}\right),\\ \mathrm{if}\;\mathrm{HMCR}\;\leq \;\mathrm{rand} \end{array}$$

(17) $$\mathrm{HMCR}=0.5\left(1-\frac{i}{\max\left(i\right)}\right),\;\mathrm{PAR}=0.05\left(1-\frac{i}{\max\left(i\right)}\right)$$

After each harmony search iteration, the newly generated HVs are compared to the existing ones in terms of their performance and, in cases where they demonstrate better performance, they replace the old HVs. After each iteration, the best-performing HV is identified. The process is repeated until the performances converge to an optimum level. For further details of the harmony search algorithm, it is suggested that the reader see reference [50].

## 3. Results and Discussions

This section presents the performances of eight different machine learning algorithms in predicting the axial load-carrying capacity of FRP-RC columns based on the experimental database of 117 samples collected from the literature [25]. The performances of these algorithms were measured using four different metrics of accuracy, the details of which have been given in Appendix B. The machine learning algorithms were ranked according to their accuracy in predicting the axial load-carrying capacity and their computational speed. The impacts of different input variables on the machine learning model output were investigated using the SHAP methodology. After the determination of the most significant input variables and their dependencies, three different equation formats were proposed for the prediction of the axial load-carrying capacity. A harmony search algorithm was utilized for the development of these equations. Finally, the performances of the proposed equations are presented.

### 3.1. Machine Learning Model Performances

The machine learning models were trained after splitting the database of experimental samples into a training set and a test set in a 90% to 10% ratio. The accuracies and computational speed of these models are presented for training and test sets separately.

**Figure 2:**
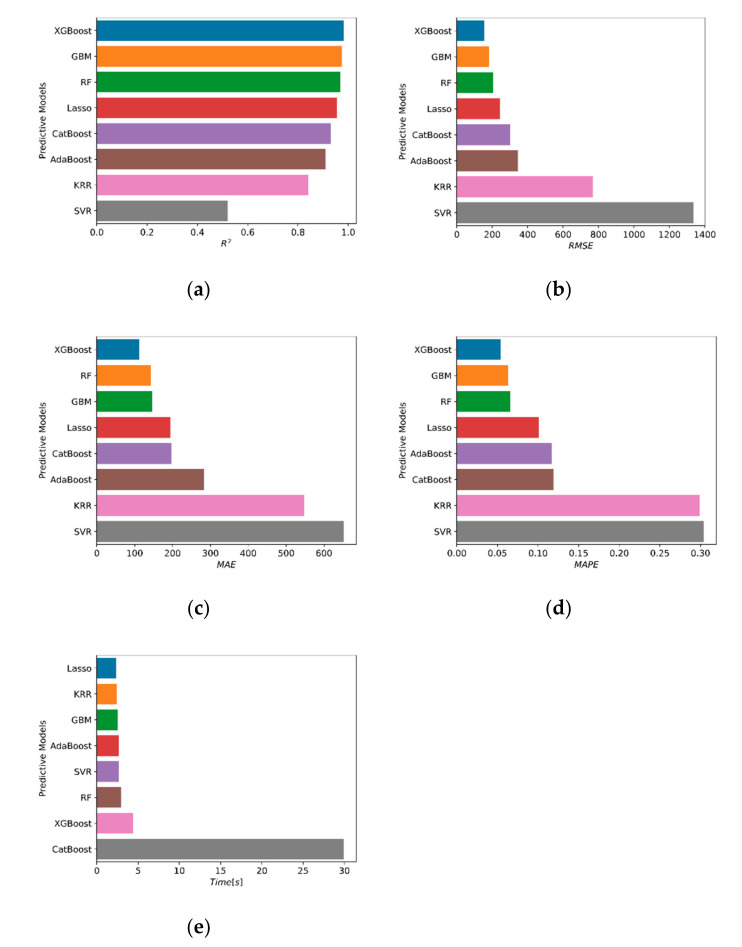
Comparison of predictive models with respect to accuracy and speed; (**a**) R^2^; (**b**) RMSE; (**c**) MAE; (**d**) MAPE; (**e**) Time [s].

**Figure 3:**
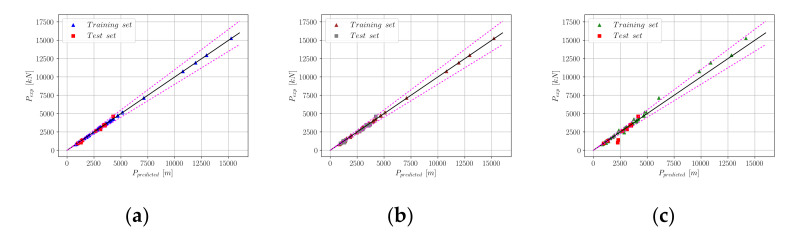

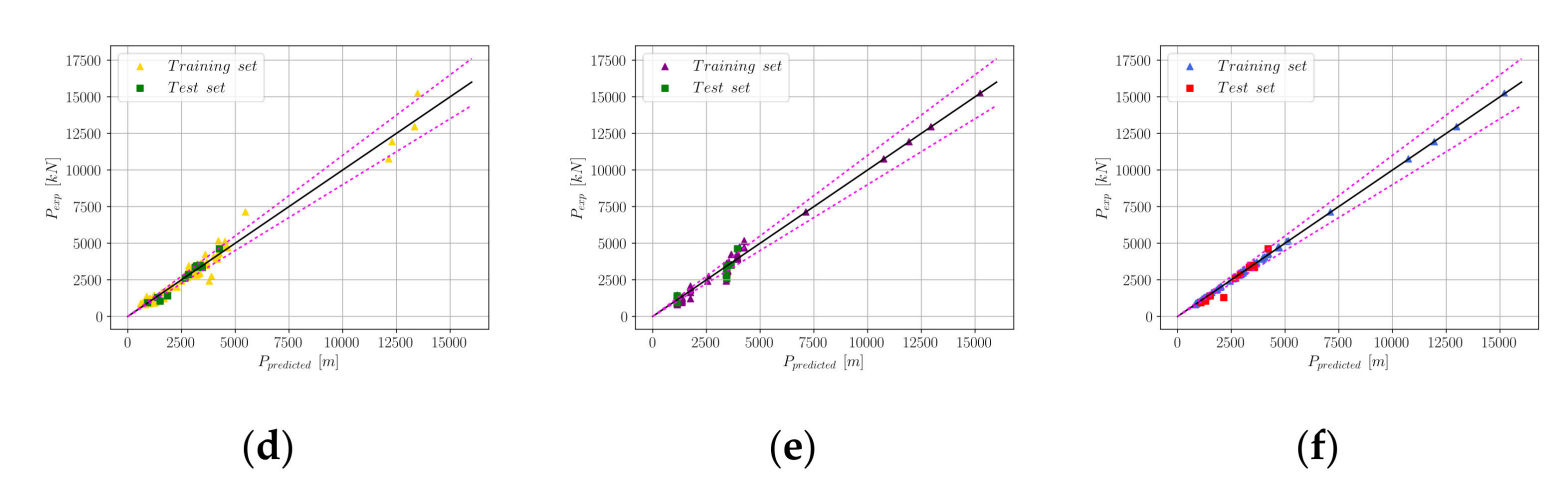
Comparison of experimental and predicted *p* values; (**a**) XGBoost; (**b**) GBM; (**c**) Random Forest; (**d**) Lasso; (**e**) AdaBoost; (**f**) CastBoost.

A list of algorithms and their accuracies with respect to four different metrics of accuracy can be found in Table 1. In Table 1, for each algorithm, the time that elapses from the beginning of the model fitting process to the end of the predictions for the test set is listed. According to Table 1, the Gradient Boosting Machine (GBM) algorithm emerges as the most efficient algorithm considering both prediction accuracy and time elapsed, since this model delivered the most accurate predictions next to XGBoost in 58% of the time. Among the other predictive models, Random Forest (RF) and Lasso Regression can be counted as models that delivered a greater than 95% R^2^ score on the test set in less than 3 s. The CatBoost algorithm was observed to take a significantly longer time with a relatively low R^2^ score on the test set compared to the top-performing models. The model performances in terms of accuracy metrics and speed of computation are visualized in Figure 2, where the horizontal axes represent the metrics of accuracy and speed. In Figure 3, the experimental axial load-carrying capacity values are plotted against the model predictions. The straight black lines in these plots represent a perfect match between the predicted and actual values, whereas the dotted red lines represent a ±10% deviation from a perfect match.

### 3.2. Application of the SHAP Algorithm

**Figure 4:**
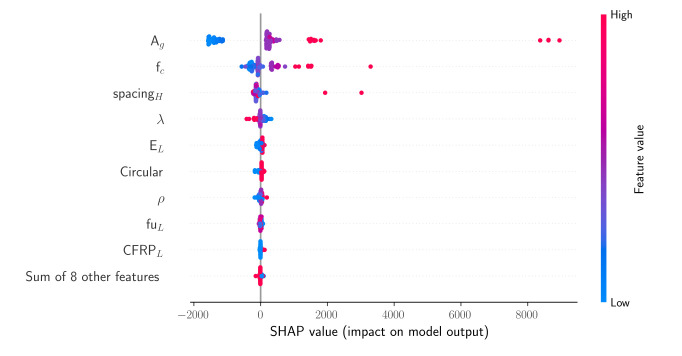
SHAP values of the design variables for the XGBoost model.

The SHAP algorithm was applied to the XGBoost model, which demonstrated the best overall performance. The SHAP summary plot is an information-intense representation of how the various design variables affect the axial load-carrying capacity. The SHAP summary plot in Figure 4 shows the SHAP values of the different variables together with a color bar that indicates whether a variable has a high or low value. Figure 4 shows the SHAP values calculated for each variable at each sample point, where every dot corresponds to a sample. Around certain SHAP values, the dots have greater concentration. For the most significant nine variables, the SHAP values were computed separately. For the remaining variables, the SHAP values were summed, and they are represented in Figure 4 as a single variable. For any sample represented in Figure 4, the color of the corresponding dot has a shade of red if a variable has a high value at this sample, and the dot has a shade of blue if the variable has a low value. Positive and negative SHAP values indicate increasing and decreasing effects on the model output, respectively. According to Figure 4, the gross cross-sectional area of the column, the concrete compressive strength, the horizontal reinforcement spacing, and the slenderness ratio (λ) have the greatest impact on the model output. Furthermore, increasing the values of Ag and *f_c_*’ also affects the prediction, whereas the opposite can be said for the slenderness ratio.

**Figure 5:**
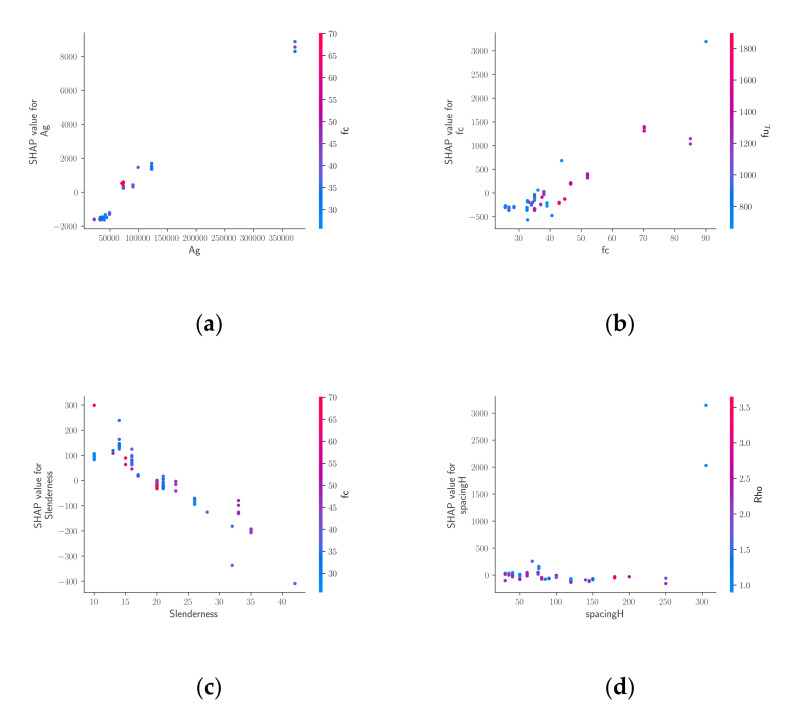
Feature dependence plots for the XGBoost model; (**a**) $A_{g}-f_{c}^{’}$; (**b**) $f_{c}^{’}-f_{\mathrm{uL}}$; (**c**) $\mathrm{Slenderness}-f_{c}^{’}$; (**d**) ${\mathrm{spacing}}_{H}-\rho$.

The feature dependence plots in Figure 5 contain information that supplements the SHAP summary plot. In Figure 5, the feature dependence plots of the four most significant design variables can be seen. For each one of these variables, the variation of another variable that is most dependent on it is presented using color. For each dot in Figure 5, a shade of red indicates a high value of the most dependent variable, whereas a shade of blue indicates a low value of the most dependent variable. The main information contained in Figure 5 is the relationship between the values of a variable and the associated SHAP values. Figure 5 shows that increasing the values of Ag and *f_c_*’ also leads to an increase in the corresponding SHAP values, which indicates an increasing effect on the model output. On the other hand, the slenderness ratio and the model output are inversely proportional. Furthermore, increasing the horizontal reinforcement spacing does not have a significant effect on the model output. The developed Python-based ML models to estimate the axial capacity of FRP-RC column can be found at https://github.com/ccakiroglu/FRPRCColumn (accessed on 4 April 2022).

### 3.3. Development of the Predictive Equations

Based on the SHAP summary plot in Figure 4 and the feature dependence plots in Figure 5, three different equation formats were proposed. The first one of these formats, given in Equation (18), consists of the linear combination of three product terms. The first one of these terms includes, besides the most important input variable $A_{g}$, the variables $f_{c}$, which is the most dependent variable on $A_{g}$, and $f_{\mathrm{uL}}$, which is the most dependent variable on $f_{c}$. The second term consists of the multiplication of the slenderness ratio λ and $f_{c}$, which is the most dependent variable on λ. Finally, the third term includes the effects of the spacing between the horizontal reinforcements and the longitudinal reinforcement ratio (ρ), which is the most dependent variable on it.

(18) $$P=a_{0}\;+\;a_{1}A_{g}^{a_{2}}f_{c}^{a_{3}}f_{\mathrm{uL}}^{a_{4}}\;+\;a_{5}\lambda^{a_{6}}f_{c}^{a_{7}}+a_{8}{\mathrm{spacing}}_{H}^{a_{9}}\rho^{a10}$$

**Figure 6:**
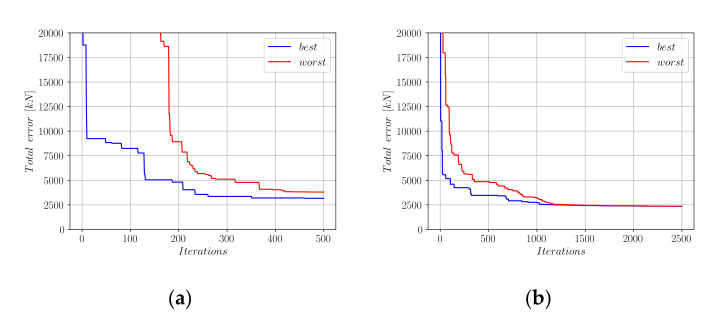
Decrease in the total error throughout the harmony search iterations (Equation (18)). (**a**) First 500 HS iterations; (**b**) First 2500 HS iterations.

An initial population of 20 candidate solution vectors was created. In this population, each solution candidate consists of a randomly generated, unique combination of the coefficients a_0_ to a_10_, where all these coefficients take values in (−1, +1). Afterward, these coefficients went through harmony search iterations. In order to train the coefficients of Equation (18), the entire database of experimental results was split into a training set and a test set in a 90% to 10% ratio. For each member of the population, the difference between the actual experimental axial load-carrying capacities (P_exp_) and the *p* values computed by Equation (18) was calculated and stored in a vector that has the length of the entire training set. As the metric that shows the accuracy of the proposed equation, the Euclidean norm of this vector was calculated. Figure 6 shows the decrease in this vector norm throughout the optimization steps.

**Figure 7:**
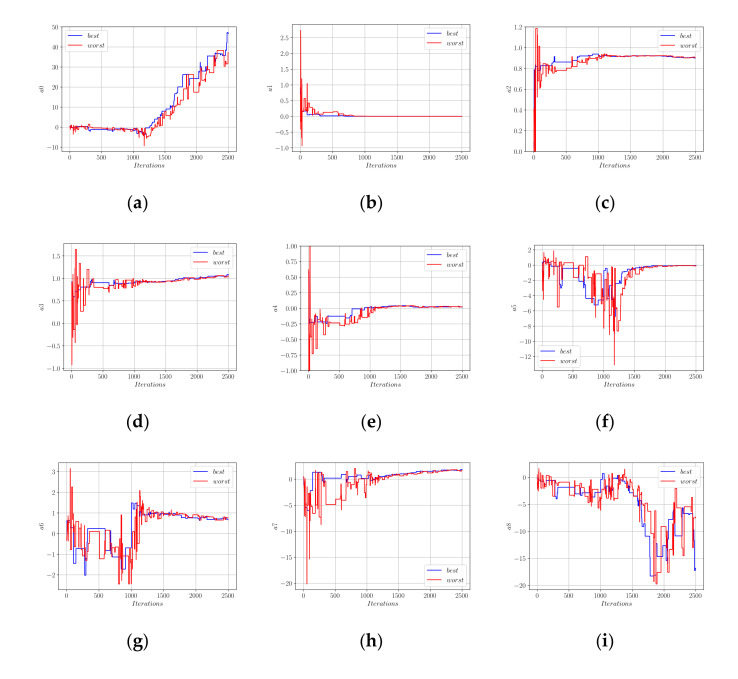

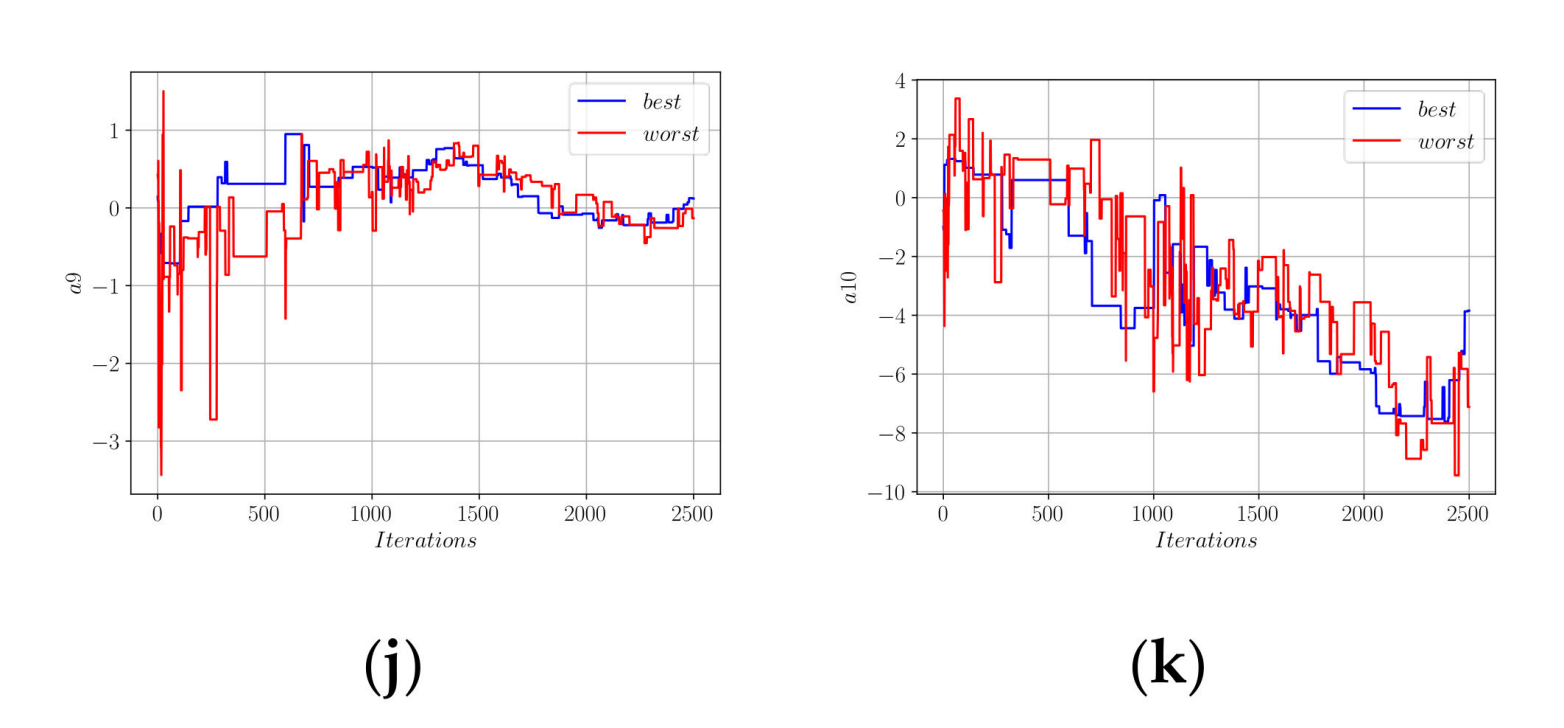
Development of the coefficients in Equation (18); (**a**) $a_{0}$; (**b**) $a_{1}$; (**c**) $a_{2}$; (**d**) $a_{3}$; (**e**) $a_{4}$; (**f**) $a_{5};$ (**g**) $a_{6}$; (**h**) $a_{7}$; (**i**) $a_{8}$; (**j**) $a_{9}$; (**k**) $a_{10}$.

In Figure 6, the performances of the best- and worst-performing solution candidates are shown with blue and red colors, respectively. Figure 6b shows that, after 2500 iterations, a convergence to the minimum total error norm could be observed. The development of the coefficients $a_{0}$ to $a_{10}$ in the optimization process are shown in Figure 7.

In Figure 7, for each coefficient, the values these coefficients took in the best- and worst-performing members of the population are plotted in blue and red colors, respectively. Using the limit values of the coefficients in Figure 7, Equation (18) can be rewritten as in Equation (19).

(19) $$P=46.75\;+\;0.00207A_{g}^{0.901}f_{c}^{1.085}f_{\mathrm{uL}}^{0.02185}\;+\;\left(-0.0493\right)\lambda^{0.676}f_{c}^{1.862}\;+\;\left(-16.84\right){\mathrm{spacing}}_{H}^{0.122}\rho^{-3.84}$$

**Figure 8:**
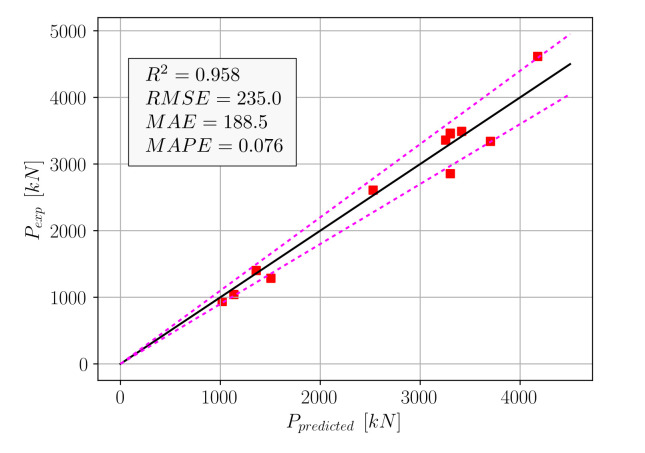
Comparison of experimental and predicted (Equation (19)) *p* values.

Using Equation (19), the *p* values were computed for the test set and compared to the actual measured values. This comparison can be seen in Figure 8, where the dotted lines indicate the ±10% deviations from an exact match.

The accuracy metrics in Figure 8 show that Equation (19) outperformed five out of the eight algorithms in Table 1. The machine learning algorithms that performed better than Equation (19) were Random Forest, XGBoost, and GBM. Although good performance could be obtained from Equation (19), a second equation format for practical use was proposed, as in Equation (20).

(20) $$P=a_{0}\;+\;a_{1}A_{g}^{a_{2}}f_{c}^{a_{3}}\;+\;a_{4}A_{g}^{a_{5}}spacing_{H}^{a_{6}}\;+\;a_{7}A_{g}^{a_{8}}\lambda^{a9}$$

In Equation (20), the number of variables being considered in the prediction and the total number of coefficients were reduced. The variables having the highest impact on the model output according to the SHAP summary plot were selected. Equation (20) consists of three product terms after *a*_0_. In each one of these terms, *A_g_* is multiplied with one of the three most significant variables after itself, according to the SHAP summary plot. The harmony search procedure was repeated for Equation (20).

**Figure 9:**
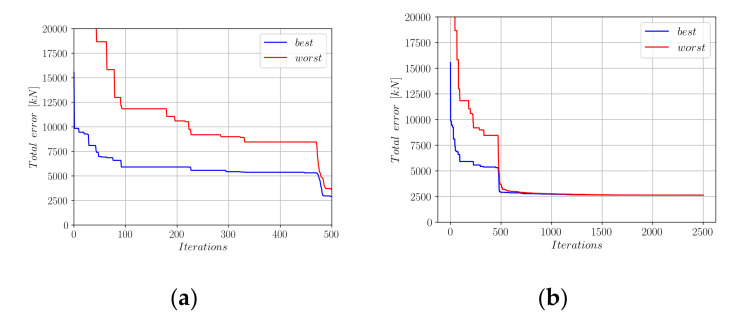
Decrease in the total error throughout the harmony search iterations (Equation (20)). (**a**) First 500 HS iterations; (**b**) First 2500 HS iterations.

**Figure 10:**
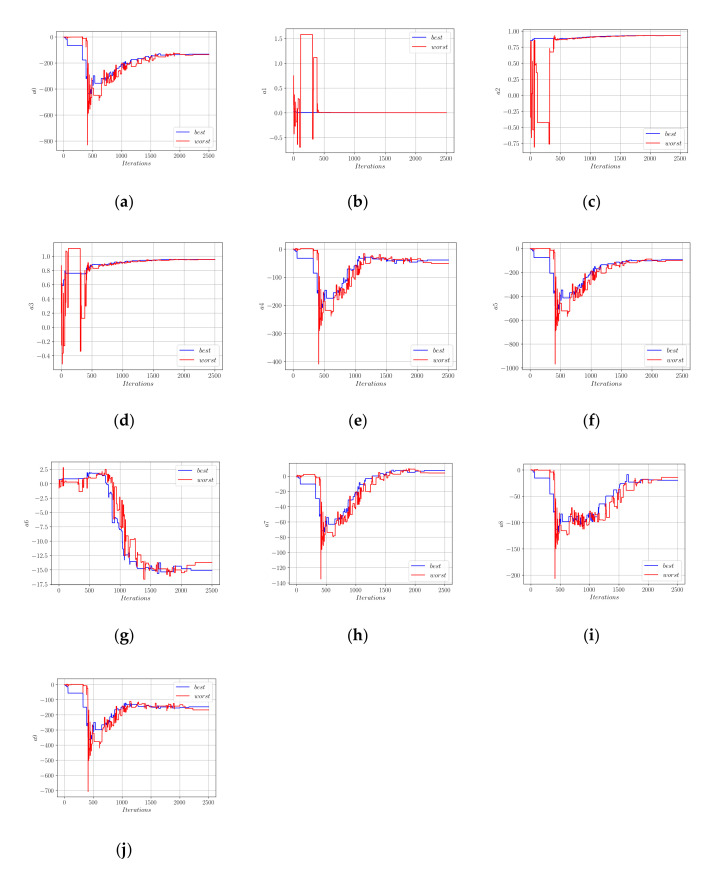
Development of the coefficients in Equation (20); (**a**) $a_{0}$; (**b**) $a_{1}$; (**c**) $a_{2}$; (**d**) $a_{3}$; (**e**) $a_{4}$; (**f**) $a_{5};$ (**g**) $a_{6}$; (**h**) $a_{7}$; (**i**) $a_{8}$; (**j**) $a_{9}$.

Figure 9a,b show the development of the error norm during the first 500 and 2500 harmony search iterations, respectively. Using the Euclidean norm of the difference vector between the actual measurements and predictions of Equation (20), a minimum error of 2639 kN could be achieved. This error norm was slightly greater than the 2336 kN that could be achieved using the Equation (18) format. Figure 10 shows the development of the coefficients *a*_0_ to *a*_9_ in Equation (20) during the harmony search process.

In both Figure 7 and Figure 10, it can be observed that the coefficients of the worst population member demonstrated much wider fluctuations than the best member. However, after a certain number of iterations, both the best and worst member coefficients converged nearly to the same values. Using the limit values of the best member coefficients, Equation (21) was obtained.

(21) $$P=-131.77\;+\;0.002608A_{g}^{0.93058}f_{c}^{0.955}\;+\;\left(-38.76\right)A_{g}^{-93.69}{\mathrm{spacing}}_{H}^{-15.07}\;+\;7.58A_{g}^{-19.54}\lambda^{-146.67}$$

The negative powers in the second and third products of Equation (21) make these terms close to zero so that Equation (21) can be used in a simplified format, as in Equation (22).

(22) $$P=-131.77\;+\;0.002608A_{g}^{0.93058}f_{c}^{0.955}$$

**Figure 11:**
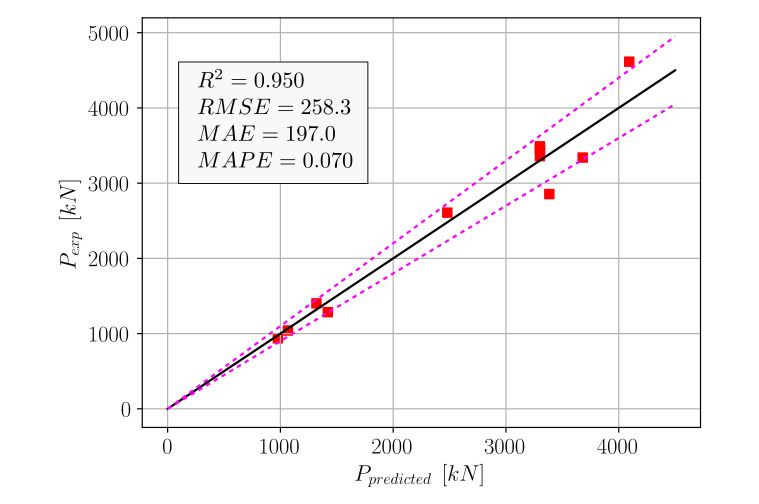
Comparison of experimental and predicted (Equation (22)) *p* values.

Figure 11 shows the comparison of the experimental axial load-carrying capacities to the *p* values predicted by Equation (22). According to the accuracy values in Figure 11, Equation (22) was able to outperform the KRR, SVR, AdaBoost, and CatBoost algorithms. A comparison of Figure 8 and Figure 11 shows that Equation (22) was able to achieve comparable performance to Equation (19) in a much simpler format. In order to eliminate the bias terms in Equations (19) and (22), a third equation format that consists of a single product term was proposed in Equation (23).

(23) $$P=a_{0}A_{g}^{a_{1}}f_{c}^{a_{2}}{\mathrm{spacing}}_{H}^{a_{3}}\lambda^{a4}\rho^{a_{5}}f_{\mathrm{uL}}^{a6}$$

**Figure 12:**
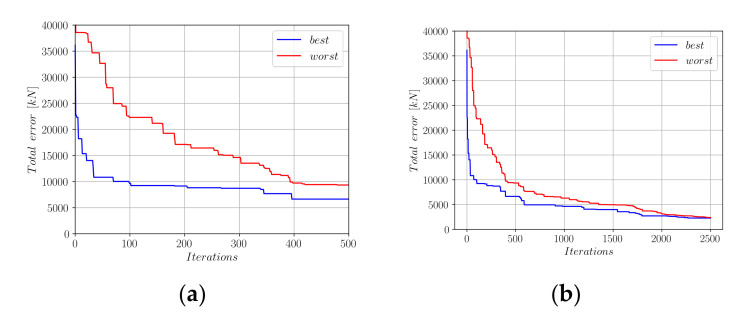
Decrease in the total error throughout the harmony search iterations (Equation (23)); (**a**) First 500 HS iterations; (**b**) First 2500 HS iterations.

Figure 12 shows the development of the error norm for the best- and worst-performing harmony vectors for the first 500 and 2500 iteration steps.

Figure 12 shows that a minimum error norm of 2286 kN could be achieved after 2500 iterations which is less than the error norms achieved through Equations (18) and (20). After inserting the limit values of the coefficients $a_{0}$ to $a_{6}$ into Equation (23), we obtain Equation (24).

(24) $$P=0.00123A_{g}^{0.9946}f_{c}^{0.9266}{\mathrm{spacing}}_{H}^{0.01124}\lambda^{-0.1474}\rho^{0.08769}f_{\mathrm{uL}}^{0.0589}$$

**Figure 13:**
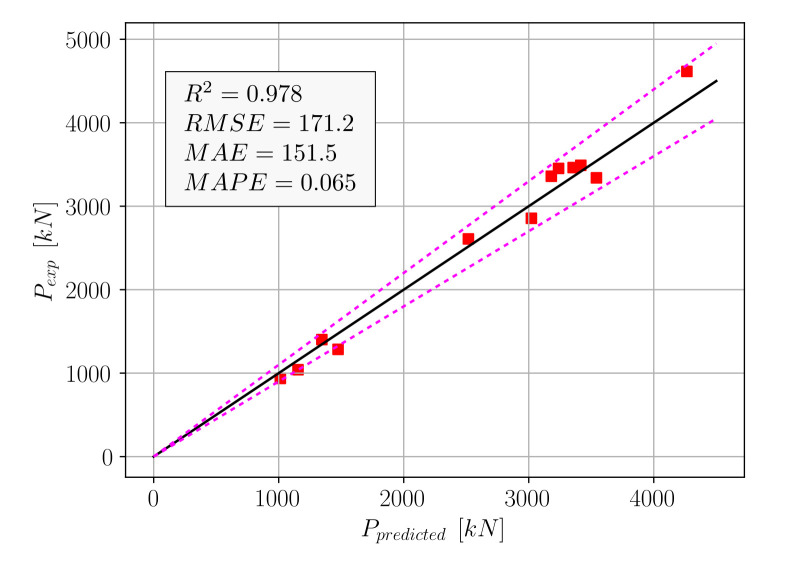
Comparison of experimental and predicted (Equation (24)) *p* values.

The performance of Equation (24) in predicting the axial load-carrying capacity has been presented in Figure 13, where the actual *p* values are plotted against the *p* values predicted by Equation (24). Finally, a list of all three equations proposed in this paper can be found in Table 2 with the corresponding R^2^ values. A comparison with the R^2^ values in Table 1 shows that Equation (24) outperformed all of the predictive models except for the XGBoost model. It should be noted that the proposed equations are data-driven, and the performance of those equations depends on the characteristics of the data used to develop the ML models. The equations are only applicable for the range of maximum and minimum values of the input parameters.

## 4. Summary and Conclusions

This work aimed to address an important gap in the research literature dealing with FRP-RC columns under concentric axial loading by applying various machine learning techniques to identify the most significant design variables affecting the axial load-carrying capacity. Particularly, ensemble learning techniques, such as Gradient Boosting Machine, Random Forest, and XGBoost, were observed to predict the axial load-carrying capacity with high accuracy. The coefficient of determination, root mean square error, mean absolute error, and mean absolute percentage error were used to quantify the accuracy of the predictions made by eight different machine learning models. Based on the predictive model with the best performance, the SHAP algorithm was utilized to identify those variables that have the greatest impact on the structural response. The gross cross-sectional area was found to have the greatest impact on the model output, followed by the concrete compressive strength, spacing of transverse reinforcement, and the slenderness ratio. Once the design variables were ranked according to their impact on the model output, in the next part of the paper, three different equation formats were proposed for the prediction of the axial load-carrying capacity as a function of the most significant design variables. These equations were optimized using the harmony search algorithm, and the equations were observed to have good accuracy. The first one of these equations predicts the axial load-carrying capacity as a function of the gross cross-sectional area, concrete compressive strength, the ultimate strength of the longitudinal reinforcements, slenderness ratio, spacing between transverse reinforcements, and the longitudinal reinforcement ratio. On the other hand, the second equation depends only on the two most significant variables, namely, the gross cross-sectional area and the compressive strength of concrete. Finally, the third equation consists of a single product term that consists of the gross cross-sectional area, the concrete compressive strength, spacing between transverse reinforcements, slenderness ratio, longitudinal reinforcement ratio, and the ultimate strength of the longitudinal reinforcements. With an R^2^ score of 0.978, the third equation performed better than the first two equations. Additionally, this equation outperformed all of the predictive machine learning models except for the XGBoost model.

The availability of closed-form equations for an accurate prediction of structural response is beneficial in engineering practice. Due to an R^2^ score of 0.95 and its simple format, the second prediction equation developed in this study is convenient for practical application. On the other hand, for more accurate predictions, the third equation can be used.

However, it should be noted that the developed equations are based on an experimental database consisting of 117 samples, and further studies in this area with larger databases are warranted. Furthermore, it should be noted that the results predicted by the developed equations are only valid within the range of the database used. Readers should keep in mind that the proposed equations are data-driven and can violate the mechanics-based capacity prediction model if misrepresented data are used. In addition to experimental studies, the databases could be further enhanced with the help of well-calibrated finite-element models. Besides increasing the size of the database used in model training, future research in this area can focus on the prediction of the axial load-carrying capacity under eccentric axial loading.

## Figures and Tables

**Table 1:** Performance comparison of different machine learning algorithms.

	R^2^	RMSE	MAE	MAPE	Time Elapsed
Train (KRR)	0.903	744	492	0.243	2.40 s
Test (KRR)	0.841	768	548	0.299	
Train (SVR)	0.522	1646	604	0.182	2.67 s
Test (SVR)	0.520	1334	652	0.304	
Train (Lasso)	0.971	399	236	0.096	2.36 s
Test (Lasso)	0.955	244	194	0.101	
Train (GBM)	0.999	57.9	44.1	0.022	2.54 s
Test (GBM)	0.975	182.9	146	0.063	
Train (AdaBoost)	0.973	384	284	0.119	2.64 s
Test (AdaBoost)	0.910	345	283	0.117	
Train (RF)	0.993	193	80.7	0.027	2.95 s
Test (RF)	0.969	204	142.4	0.066	
Train (XGBoost)	0.999	22.3	7.5	0.003	4.41 s
Test (XGBoost)	0.982	153.8	112.7	0.054	
Train (CatBoost)	0.999	35.7	28.9	0.013	29.92 s
Test (CatBoost)	0.931	301	197.4	0.119	

**Table 2:** Proposed equations.

Equation	R^2^
$P=46.75\;+\;0.00207A_{g}^{0.901}f_{c}^{1.085}f_{\mathrm{uL}}^{0.02185}\;+\;\left(-0.0493\right)\lambda^{0.676}f_{c}^{1.862}\;+\;\left(-16.84\right){\mathrm{spacing}}_{H}^{0.122}\rho^{-3.84}$	0.958
$P=-131.77\;+\;0.002608A_{g}^{0.93058}f_{c}^{0.955}$	0.950
$P=0.00123A_{g}^{0.9946}f_{c}^{0.9266}{\mathrm{spacing}}_{H}^{0.01124}\lambda^{-0.1474}\rho^{0.08769}f_{\mathrm{uL}}^{0.0589}$	0.978

## Data Availability

Data sharing is not applicable for this paper.

## Appendix A

**Nomenclature**		XGBoost	Extreme gradient boosting
		KRR	Kernel ridge regression
FRP	Fiber-reinforced polymer	SVR	Support vector regression
GFRP	Glass fiber-reinforced polymer	CatBoost	Categorical gradient boosting
CFRP	Carbon fiber-reinforced polymer	AdaBoost	Adaptive boosting
Ag	Gross cross-sectional area	GBM	Gradient boosting machine
spacing_H_	Spacing between transverse reinforcements	SHAP	Shapley additive explanations
Pexp	Experimental axial load-carrying capacity	*f_c_*’	Concrete compressive strength
MAE	Mean average error	MAPE	Mean average percentage error
RMSE	Root mean square error	R^2^	Coefficient of determination
HMS	Harmony memory size	HV	Harmony vector
HMCR	Harmony memory consideration rate	PAR	Pitch adjustment rate
λ	Slenderness ratio	ρ	Longitudinal reinforcement ratio
fuL	Ultimate strength of the longitudinal reinforcements	*E_FRP_*	Modulus of elasticity of the longitudinal reinforcements

## Appendix B

Definitions of the accuracy metrics used in this paper:

(A1) $$R^{2}=1-\frac{\sum_{i=1}^{N}{\left(P_{i}-{\hat{P}}_{i}\right)}^{2}}{\sum_{i=1}^{N}{\left(P_{i}-P_{\mathrm{mean}}\right)}^{2}}$$

(A2) $$\mathrm{RMSE}=\sqrt{\frac{\sum_{i=1}^{N}{\left(P_{i}-{\hat{P}}_{i}\right)}^{2}}{N}}$$

(A3) $$\mathrm{MAPE}=\frac{100\sum_{i=1}^{N}\frac{\left|P_{i}-P_{i}\right|}{\left|P_{i}\right|}}{N}$$

(A4) $$\mathrm{MAE}=\frac{\sum_{i=1}^{N}\left|P_{i}-P_{i}\right|}{N}$$
